# The complete plastome of *Polytrichum commune* Hedw. (Polytrichaceae, Bryophyta)

**DOI:** 10.1080/23802359.2021.1927223

**Published:** 2021-05-13

**Authors:** Xin-Jie Jin, Rui-Liang Zhu

**Affiliations:** Bryology Laboratory, School of Life Sciences, East China Normal University, Shanghai, China

**Keywords:** Chloroplast genome, hair-cap moss, phylogenomics, Polytrichopsida

## Abstract

*Polytrichum commune*, one of hair-cap mosses, is the type species of the genus *Polytrichum* Hedw. (Polytrichaceae). Here we present its complete plastome. The plastome of *P. commune* is successfully assembled from raw reads sequenced by HiSeq X ten system. Its total length is 126,323 bp consisting of four regions: large single copy (LSC) region (88,070 bp), small single copy (SSC) region (16,717 bp), and inverted repeats (IRs; 9,680 bp per each). It contains 128 genes (84 coding genes, eight rRNAs, and 36 tRNAs); nine genes (four rRNAs and five tRNAs) are duplicated in IR regions. The overall GC content is 28.9% and in the LSC, SSC and IR regions is 26.1%, 25.1%, and 45.5%, respectively. This plastome is an important sequence resource for further studies on the class Polytrichopsida.

*Polytrichum commune* Hedw., commonly known as a hair-cap moss, has been used as a traditional Chinese medicine as it has anticancer activity (Fu et al. 2009; Yuan et al. 2015). It shows clear differentiation of water-conducting tissue (hadrom and leptom), which is analogous to vascular tissue (xylem and phloem) of higher plants (Eschrich and Steiner 1968). *Polytrichum* is distinguished from the allied genus *Pogonatum* P. Beauv. by capsules with stomae (Smith and Gary 2007). Recent molecular phylogenetic studies presented that *P. commune* is a crown group of the family Polytrichaceae (Hyvönen et al. 2004), which is the sole member of the class Polytrichopsida. Till now, *Pogonatum inflexum* (Lindb.) Sande Lac. is the only available complete chloroplast genome of Polytrichopsida in GenBank. *Polytrichum juniperinum* Hedw. and *Polytrichum strictum* Menzies ex Brid. have only partial chloroplast genome data (de Freitas et al. 2018). Here, we present the plastome of *P. commune* as a first complete plastome of *Polytrichum*, the type genus of Polytrichaceae.

*Polytrichum commune* was collected in the tea farm of Fengyang Mountain, Zhejiang, China (27°52′46″N, 119°10′45″E). The specimen was deposited at the herbarium of East China Normal University (HSNU, http://museum.ecnu.edu.cn/; Rui-Liang Zhu, rlzhu@bio.ecnu.edu.cn) under the voucher number Zhu & Zhang 20200723-18. DNA was extracted using DNA Plantzol Reagent (Hangzhou Lifefeng Biotechnology Co., LTD). Genome sequencing was performed using HiSeq X ten system at BGI (Shenzhen), China, and *de novo* assembly was done by the GetOrganelle pipeline (Jin et al. 2020). The raw data were assembled using GetOranelle version 1.5.1 with the command get_organelles_reads.py. The command lines are as follows: get_organelle_reads.py −1 forward.fq −2 reverse.fq -o plastome_output -R 15 -k 21,45,65,85,105 -F plant_cp. The detailed steps to use getoranelle are shown on the website (https://github.com/Kinggerm/GetOrganelle). Geneious version 11.0.3 (Kearse et al. 2012) was used for plastome annotation, with *Diphyscium foliosum* (Hedw.) D. Mohr plastome (MN496311, Bell et al. 2020) as reference. CPGAVAS2 was used to further verify the tRNA genes (Shi et al. 2019).

The plastome of *P. commune* (GenBank accession MW528408) is 126,323 bp long (GC ratio is 28.9%) and has four subregions: 88,070 bp of large single copy (26.1%) and 16,717 bp of small single copy (25.1%) regions separated by 9,680 bp of inverted repeat (IR; 45.5%). It is longer than the sister species *Pogonatum inflexum* (MK131349, 125,415 bp) and contains 128 genes (84 protein-coding genes, eight rRNAs, and 36 tRNAs) and nine genes (four rRNAs and five tRNAs) duplicated in IR regions.

Sixteen complete chloroplast genomes (Figure 1) including *P. commune* were used for Bayesian Inference (BI, 2,000,000 generations, sampled every 1000 generations) and Maximum Likelihood (ML, bootstrap repeat is 1000) phylogenic trees using MRBAYES v3.2.7 (Ronquist and Huelsenbeck 2003) and IQ-TREE (Nguyen et al. 2015), respectively, after aligning whole plastome sequences using MAFFT v7.149b (Katoh and Standley 2013).

**Figure 1.**
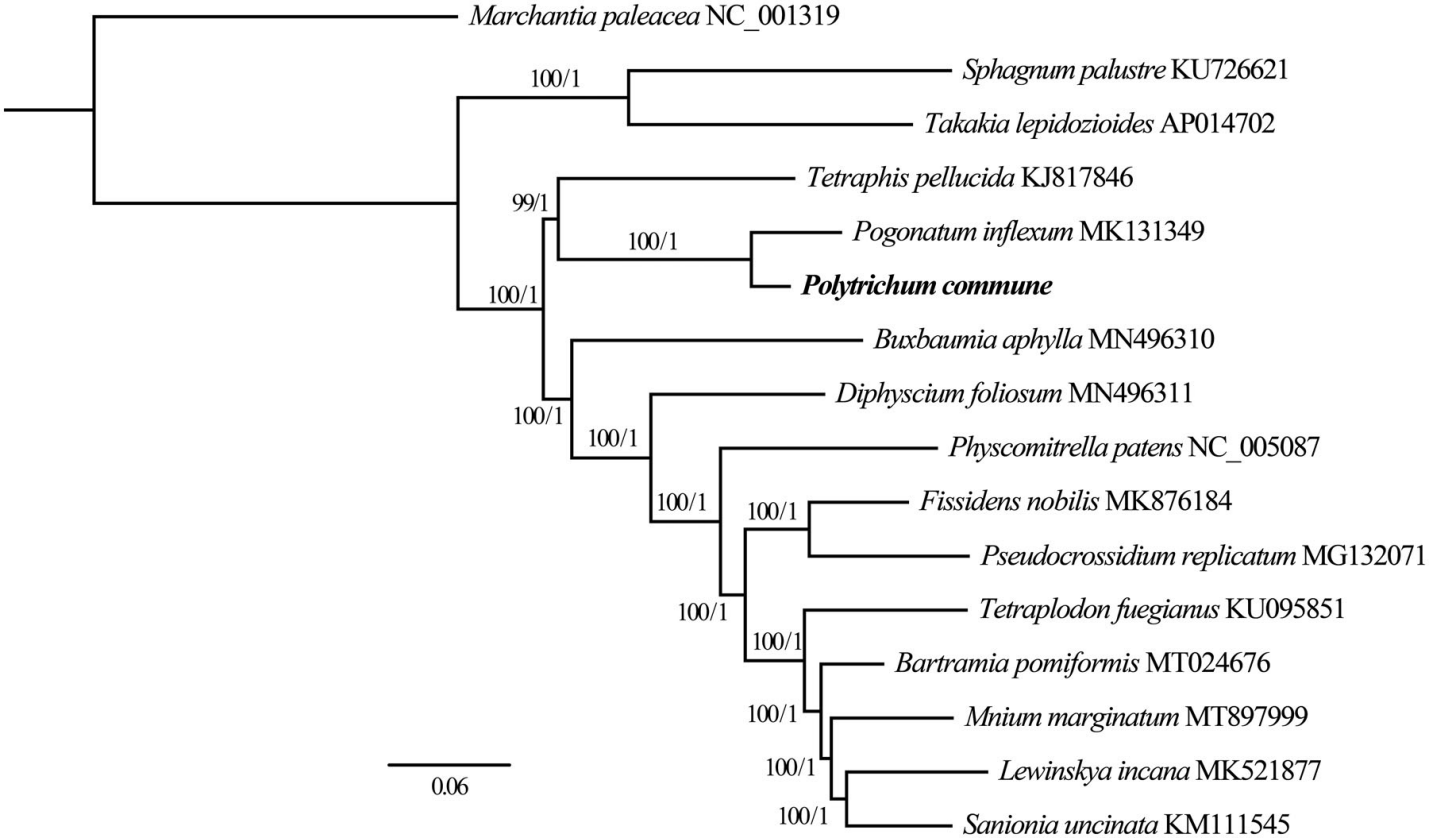
Maximum Likelihood (ML) and Bayesian Inference (BI) phylogenetic tree of 16 complete chloroplast genomes: *Polytrichum commune* (MW528408, in this study)*, Bartramia pomiformis* (MT024676), *Buxbaumia aphylla* (MN496310), *Diphyscium foliosum* (MN496311), *Fissidens nobilis* (MK876184), *Lewinskya incana* (MK521877), *Marchantia paleacea* (NC_001319), *Mnium marginatum* (MT897999), *Pogonatum inflexum* (MK131349), *Physcomitrella patens* (NC_005087), *Pseudocrossidium replicatum* (MG132071), *Sanionia uncinata* (KM111545), *Sphagnum palustre* (KU726621), *Takakia lepidozioides* (AP014702), *Tetraphis pellucida* (KJ817846) and *Tetraplodon fuegianus* (KU095851). The ingroup consisted of 15 moss species representing 14 orders and five classes and *Marchantia paleacea* (NC_001319) as an outgroup. Phylogenetic tree was drawn based on ML tree. The numbers above branches indicate bootstrap values (BS) and Bayesian Posterior Probabilities (PP).

Phylogenetic trees show that class Polytrichopsida (*P. commune* and *P. inflexum*) is sister to class Tetraphidopsida (*Tetraphis pellucida* Hedw.), which is in accordance with previous studies (Volkmar and Knoop 2010; Liu et al. 2019) (Figure 1). In addition, a basal clade was formed by *Sphagnum palustre* L. (Shaw et al. 2016) and *Takakia lepidozioides* S. Hatt. and Inoue (AP014702), which is same as the result of Cox et al. (2004) and Qiu et al. (2006), but is incongruent with Liu et al. (2019). In summary, this suggests that additional bryophyte chloroplast genomes are needed to elucidate the phylogenetic relationships of these species. With the help of next generation sequencing technology, more and more plastome sequences of mosses will be published in the near future, which will allow us to have a better understanding of their phylogenetic relationships.

## Data Availability

The genome sequence data of *Polytrichum commune* that support the findings of this study are openly available in GenBank of NCBI at (https://www.ncbi.nlm.nih.gov/) under the accession no. MW528408. The associated BioProject, Sequence Read Archive (SRA), and Biosample numbers are PRJNA698729, SRR13608611, and SAMN17734892, respectively.
